# Effects of Genotype and Sex on Carcass Traits, Myosin Heavy Chain Isoforms and Meat Characteristics of Pigs

**DOI:** 10.3390/ani15172535

**Published:** 2025-08-28

**Authors:** Chanporn Chaosap, Wuttikorn Buajoom, Numfon Pothising, Chananya Kongtasorn, Kazeem D. Adeyemi

**Affiliations:** 1 Department of Agricultural Education, Faculty of Industrial Education and Technology, King Monkut’s Institute of Technology Ladkrabang, Bangkok 10520, Thailand; wbuajoom@gmail.com; 2 Department of Animal Production Technology and Fisheries, Faculty of Agricultural Technology, King Monkut’s Institute of Technology Ladkrabang, Bangkok 10520, Thailand; numfont.j@gmail.com; 3 Department of Research and Development, Betagro Hybrid International Co., Ltd., Bangkok 10210, Thailand; chananyak@betagro.com; 4 Department of Animal Production, Faculty of Agriculture, University of Ilorin, Ilorin PMB 1515, Nigeria; kazyadeyemi@gmail.com

**Keywords:** *CAPN1*, intramuscular fat, purge loss, shear force, inosine monophosphate

## Abstract

This study investigated the effects of genotype and sex on carcass traits, myosin heavy chain (MyHC) isoforms, and meat quality in pigs raised under Thai commercial conditions. Fifty pigs (25 barrows and 25 gilts) from five genotypes—purebred Duroc (D), LWLR1 (Large White_old × Landrace_old), LWLR2 (Large White_new × Landrace_new), DLWLR1 (Duroc × LWLR1), and DLWLR2 (Duroc × LWLR2)—were slaughtered at ~110 kg body weight. LWLR1 is traditional maternal line, whereas LWLR2 is a newly selected line for higher intramuscular fat (IMF). Significant genotype × sex interactions were observed for dressing percentage, carcass length, bone percentage, purge loss, pH_24_, and L* (lightness) (*p* < 0.05). The *Longissimus lumborum* (LL) muscle of Duroc pigs showed the lowest pH_45_ and shear force but highest IMF, whereas LWLR1 pigs exhibited higher crude protein, polyunsaturated fatty acids, and flavor-enhancing nucleotides. The LL muscle of LWLR2 and DLWLR2 pigs showed greater expression of MyHC I and IIA, as well as calpain 1 (*CAPN1*), calpain 2 (*CAPN2*), and calpastatin (*CAST*) genes. In contrast, Duroc pigs had the highest MyHC IIX and lowest IIB expression, opposite to patterns in LWLR1 and DLWLR1. Principal component analysis revealed distinct genotype-based clustering, emphasizing the influence of maternal lineage on pork quality and offering valuable insights for genetic selection strategies.

## Simple Summary

This study examined how pig genotype and sex affect carcass traits, muscle fiber composition, and meat quality in commercial Thai pig production. Five genotypes, including purebred Duroc, traditional and newly selected maternal lines, and their crossbreds, were evaluated. Results showed that genotype had a stronger impact than sex on meat characteristics. Duroc pigs had the most intramuscular fat and tender meat, while traditional maternal-line pigs had higher protein and healthy fats. The new maternal line and its crossbreed showed better water-holding capacity and favorable muscle traits. These findings suggest that selecting the right maternal lines can help improve pork quality—such as tenderness, flavor, and nutritional value—supporting breeding strategies that align with consumer preferences in tropical regions.

## 1. Introduction

The meat consumption pattern in Thailand reflects the country’s diverse culinary heritage and socio-economic factors [1,2]. Meat, especially pork, chicken and beef, plays a central role in Thai’s cuisine [2]. Over the years, economic growth, urbanization, and changing dietary habits have significantly influenced meat consumption trends in Thailand [3,4]. Pork accounts for a significant share of total meat consumption in Thailand, frequently exceeding that of chicken and beef [5,6,7]. This dominance is partly due to pork’s versatility in Thai cuisine and its integration into everyday meals [5,7].

The popularity of pork in Thailand has driven the demand for more efficient production methods, leading to an increased reliance on commercial pig farming [8,9]. The use of exotic pig breeds in these commercial farms has been increasing over the years, driven by the demand for high-quality pork and specific traits such as growth rate, feed efficiency, and meat quality [9,10]. Exotic breeds like Duroc, Landrace and Large white, as well as their crosses are preferred for their desirable features, which contribute to better production outcomes compared to indigenous breeds [9]. However, the meat quality of those exotic breeds and their crosses within the context of Thai culinary and consumer preferences is not well documented.

Both genotype and sex are critical factors affecting growth performance, carcass trait, and pork quality [11,12,13]. Variations in genotype, particularly among crossbred pigs with different maternal lines, can lead to substantial differences in muscle composition, fat deposition, and sensory attributes of meat. Likewise, sex significantly influences muscle growth and fat deposition through hormonal regulation, which in turn affects meat quality [14,15]. However, the specific effects of genotype and sex on pork quality in exotic pig breeds and their crossbreds under Thai production conditions remain inadequately documented.

Understanding the effects of genotype and sex on pork quality is crucial for developing effective breeding strategies, differentiating pork products, and increasing market competitiveness. However, limited data are available on these factors under Thai production conditions. This study aimed to evaluate the effects of genotype and sex on carcass traits, muscle fiber composition, and meat quality in pigs. The breeding scheme involved crossing a purebred Duroc sire line with two maternal lines (Large White × Landrace): one representing an established commercial genotype and the other a newly developed line selected for increased intramuscular fat content. This design enabled a comprehensive assessment of maternal genetic contributions to offspring performance and meat quality. The study included gilts and castrated males from five genetic groups: purebred Duroc, two-way crossbred dams, and their three-way crossbred offspring.

## 2. Materials and Methods

### 2.1. Animals and Muscle Sampling

A total of 50 pigs (25 gilts and 25 barrows) were used in the study, with 10 pigs from each genotype (n = 5 females; n = 5 castrated males). The five genetic groups included Duroc × Duroc (D), Large White_old × Landrace_old (LWLR1), Large White_new × Landrace_new (LWLR2), Duroc × LWLR1 (DLWLR1), and Duroc × LWLR2 (DLWLR2). All pigs were raised to a market weight of approximately 110 kg before slaughter. The LWLR1 maternal line represents a traditional genetic line commonly used in commercial pig production, whereas LWLR2 is a recently developed line selected for enhanced intramuscular fat (marbling) to support premium pork production.

All pigs were reared under uniform housing and management conditions and fed the same commercial grower–finisher diets (Betagro Group, Bangkok, Thailand) with ad libitum access to feed and water. The diet was provided in two phases: grower (20–60 kg; 18% crude protein) and finisher (60 kg to market weight; 16% crude protein). At a live weight of approximately 110 kg, they were transported by truck from farms located within two hours of the slaughter facility. Pigs underwent a total of 12 h of fasting, including approximately 2.5 h of rest upon arrival prior to slaughter. Pigs were weighed to record slaughter weight and humanely slaughtered in a commercial facility.

After stunning with 400 volts and 1.5 amps for 3 s, pigs were bled and processed by scalding, dehairing, and evisceration. Carcasses were split after head removal, and hot weight and length were measured before chilling. The initial pH (pH_45_) was taken 45 min postmortem from the left *Longissimus lumborum* (LL) muscle between the 10th and 11th ribs. Following 24 h chilling at approximately 1 °C, cold carcass weight and pH_24_ were measured. Purge loss was estimated by subtracting cold from hot carcass weight. The right half was divided into picnic, butt, ham, loin, and belly. The first four cuts were weighed and dissected into lean, fat (subcutaneous, intermuscular, visceral), and bone. Each carcass component was calculated as a proportion of the cold carcass weight.

Approximately 100 g of LL muscle was collected from the left side between the 13th and 14th ribs at 1.5 h postmortem, immediately frozen in liquid nitrogen, and stored at –80 °C for molecular and biochemical assays, including gene expression analysis, ribonucleotide quantification, and fatty acid profiling. At 24 h postmortem, a 10 cm segment of the left LL muscle was excised and divided into three 3 cm portions. One portion was vacuum-sealed and stored at –20 °C for subsequent determination of sarcomere length, fiber diameter, and chemical composition. The remaining two portions were stored at 2 °C for 1 and 5 days, respectively, and then frozen at –20 °C for evaluation of physical meat quality traits.

### 2.2. Determination of Gene Expression

#### 2.2.1. RNA Extraction, cDNA Synthesis, and Quantitative Polymerase Chain Reaction (qPCR) Analysis

Total RNA was extracted from *longissimus lumborum* samples using TRIzol reagent according to the manufacturer’s instructions (Invitrogen, Paisley, UK). After deoxyribonuclease (DNase) treatment, 0.5 µg of RNA was reverse-transcribed into first-strand cDNA using random primers and Moloney murine leukemia virus reverse transcriptase in a 20 µL reaction volume (Thermo Scientific, Waltham, MA, USA). Only RNA preparations with A260/280 ratios between 1.8 and 2.0 were used, and integrity was verified by agarose gel electrophoresis. Primers employed for quantitative gene analysis are listed in Table 1. qPCR was performed with SYBR Green dye on a CFX96 Real-Time PCR Detection System (Bio-Rad, USA). Primer specificity was confirmed by single-peak melt curves, and amplification efficiencies ranged from 90% to 110%.

#### 2.2.2. Myosin Heavy Chain Isoforms Expression

First-strand cDNA from each LL muscle sample was diluted 1:5 and pooled per group to generate a standard curve dilution series. Individual samples were further diluted 1:4 for gene expression analysis. The reaction mix contained 3.5 µL cDNA, 0.4 µL of each primer, and 5 µL SYBR Green PCR Master Mix (SensiFast™, Bioline, London, UK). Reactions were run in duplicate on a Bio-Rad CFX96 (Bio-Rad, Hercules, CA, USA) with cycling at 95 °C for 2 min, then 40 cycles of 95 °C for 5 s and 55 °C for 15 s, with real-time fluorescence detection. Quantification cycle (Cq) values were calculated using Bio-Rad CFX Manager 3.1 (Bio-Rad Laboratories).

Relative standard curves were constructed for each isoform by plotting Cq values against the log_10_ of RNA equivalents. PCR efficiency (E) was calculated as E = 10^(–1/slope)^ − 1 [19]. The relative expression ratio (rER) was calculated as

(1) $$rER=\frac{{\left[1+E(MyHC\;target\;gene)\right]}^{-Cq(MyHC\;target\;gene)}}{{\left[1+E(MyHC\;control\;gene)\right]}^{-Cq(MyHC\;control\;gene)}}$$

Any primer set could serve as the reference gene, with its rER normalized to 1. Total rER values were summed, and the relative expression of each isoform was expressed as a percentage of total adult MyHC (Myosin Heavy Chain) expression [20].

#### 2.2.3. Calpain System Gene Expression Analysis

Total cDNA was diluted 1:4, and a pooled cDNA sample was generated for each group, followed by a dilution series used to construct a standard curve. Individual cDNA samples were further diluted 1:5 for gene expression analysis. Primer sets for the calpain 1 (*CAPN1*), calpain 2 (*CAPN2*), and calpatatin (*CAST*) genes were used for amplification, along with the reference gene Glyceraldehyde-3-phosphate dehydrogenase (*GAPDH*), as shown in Table 1.

The real-time PCR mix included 3.5 μL cDNA, 0.4 μL each of forward and reverse primers, and 5 μL SYBR Green PCR Master Mix (SensiFast™, Bioline, London, UK). Reactions were run in duplicate on a Bio-Rad CFX96 system (Bio-Rad, Hercules, CA, USA) with cycling at 95 °C for 2 min, then 40 cycles of 95 °C for 5 s and 55 °C for 15 s, with real-time fluorescence detection. Melting curve analysis was performed using CFX Manager™ (Bio-Rad) to verify specificity. Expression of *CAPN1*, *CAPN2*, and *CAST* was normalized to *GAPDH*.

### 2.3. Meat Quality Analysis

#### 2.3.1. pH

The pH of muscle samples was measured in duplicate at 45 min and 24 h postmortem using a pH meter with a spear glass electrode (model SG2-ELK Seven Go™, Mettler Toledo International Inc., Giessen, Germany).

#### 2.3.2. Color

The muscle sample color was measured using Commission Internationale de l’Éclairage (CIE) color values: L* (lightness), a* (redness), and b* (yellowness). Measurements were taken at two different locations on the muscle surface using a spectrophotometer with a 2.54 cm aperture (MiniScan EZ 45/0 LAV, D65 illumination, 10° observer, Hunter Associates Laboratory Inc., Reston, VA, USA).

#### 2.3.3. Drip Loss

To determine drip loss in duplicate, a 1.5 cm-thick muscle sample was weighed, placed in a plastic bag, and suspended from a hook for 48 h at 4 °C. Afterward, the sample was carefully wiped and weighed again. Drip loss was calculated as the percentage of the original muscle weight.

#### 2.3.4. Cooking Loss and Shear Force

To determine cooking loss in duplicate, muscle samples were weighed, vacuum-sealed, and cooked in an 80 °C water bath until the internal temperature reached 70 °C. After cooling under running tap water for 30 min, samples were reweighed. Cooking loss was calculated as a percentage of the initial weight.

For shear force analysis, cooked samples were cut into eight pieces (1 × 2 × 1 cm) parallel to the muscle fibers and sheared perpendicular to fiber direction using a Warner-Bratzler blade on a Texture Analyzer (EZ-SX, Shimadzu, Tokyo, Japan) equipped with a 50 kg load cell and set at 50 mm/min crosshead speed [21].

#### 2.3.5. Muscle Fiber Diameter and Sarcomere Length

For muscle fiber diameter evaluation, LL muscle was fixed in 10% formalin for 48 h, blended with 50 mL of 0.9% NaCl, and analyzed for fiber diameter using 200 randomly selected fibers under a 4× microscope with a Dino-Eye camera and Dino Capture 2.0 software (AnMo Electronics Corp., Taiwan, China). To measure sarcomere length, the helium-neon laser diffraction technique described by Cross et al. [22] was employed, with 30 sarcomeres evaluated per sample to obtain an average value.

### 2.4. Chemical Composition

Using Association of Official Analytical Chemists (AOAC) [23] methods, moisture, crude protein, ash, and ether extract were determined in meat samples. Moisture content was measured by drying at 105 °C for 12 h, followed by cooling in a desiccator prior to weighing. Nitrogen content was quantified by the micro Kjeldahl technique and converted to crude protein by multiplying by 6.25. Ether extract was obtained via petroleum ether extraction in a Soxhlet system, and ash was measured after ashing samples at 550 °C for 6 h in a muffle furnace.

### 2.5. Fatty Acid Composition

Meat sample lipids were extracted using chloroform-methanol (2:1, *v*/*v*), then transmethylated to fatty acid methyl esters (FAMEs) following AOAC protocols [23]. Methylnonadecanoate (C19:0) was used as an internal standard. FAMEs were analyzed by gas chromatography (Agilent 7890B, Santa Clara, CA, USA) equipped with a 100 m fused silica capillary column (SPTM-2560, Supelco, Bellefonte, PA, USA). A sample volume of 1 µL was injected for each analysis. The total analysis time was approximately 60 min. The conditions for gas chromatography were as follows: temperature program: starting temperature 60 °C, followed by an increase of 20 °C/min to 170 °C, 5 °C/min to 220 °C, and 2 °C/min to 240 °C; carrier gas, He; split ratio, 10:1. Fatty acid methyl esters (FAMEs) were identified and quantified using a standard mixture of 37-component FAME mix (Supelco, Sigma-Aldrich, Bellefonte, PA, USA). Peaks were confirmed by comparing retention times with those of the analytical standards.

### 2.6. Ribonucleotides

Following Chaosap et al. [24], 1 g of pulverized breast muscle was homogenized in 6 mL of cold 0.6 M perchloric acid (23,000× *g*, 10 s), kept on ice for 15 min, then neutralized with 5.4 mL of 0.8 M KOH and 0.25 mL KH_2_PO_4_ buffer. The pH was adjusted to 7, and the volume brought to 15 mL with HPLC-grade water. After centrifugation (10,000× *g*, 10 min, 4 °C), 1 mL of the supernatant was frozen at –80 °C and analyzed for ribonucleotide content within one month. Ribonucleotides (IMP, inosine, hypoxanthine, GMP) were quantified by HPLC (Chromaster, Hitachi, Tokyo, Japan) using a TSK Gel Amide-80 column (Tosoh, Tokyo, Japan) with UV detection at 210 nm and an acetonitrile:KH_2_PO_4_ (70:30) mobile phase, based on external standard calibration curves.

### 2.7. Statistical Analysis

Data were analyzed using a two-way ANOVA with the general linear model procedure (Proc GLM, SAS Institute Inc., Cary, NC, USA). The model included the fixed effects of genotype, sex), and their interaction. Least-squares means were compared using the PDIFF option. Normality of the data was assessed using the Shapiro–Wilk test, and homogeneity of variance was checked using Levene’s test. Values of *p* < 0.05 were considered statistically significant. The model included terms according to the following equation:

Y_ijk_ = µ + G_i_ + S_j_ + G_i_ × S_j_ + €_ijk_, (2)

where Y_ijk_ was the dependent variable, µ was the overall mean, G_i_ was the fixed effect of pig genotypes or groups (_j_ = 1, 2, 3, 4, 5), S_j_ was the fix effect of pig sex: female or castrated males, G_i_ × S_j_ was the interaction between genotype and sex, and €_ijk_ was the residual error.

Principal component analysis biplot of the parameters related to meat quality traits, chemical composition, and expression of myosin heavy chain isoforms and calpains genes across different pig genotypes were performed in XLSTAT software version 2021.5 (Addinsoft, New York, NY, USA) using standardized variables, retaining components with eigenvalues >1. The proportion of explained variance and major contributing variables are reported in the Results.

## 3. Results

### 3.1. Carcass Traits

**Figure 1:**
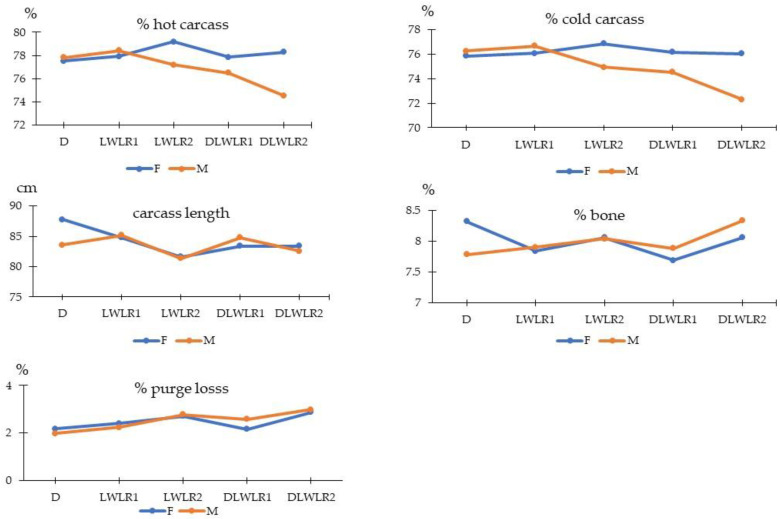
Interaction between genotypes and sexes on carcass traits in pigs. D (Duroc × Duroc); LWLR1 (Large White_old × Landrace_old); LWLR2 (Large White_new × Landrace_new); DLWLR1 (Duroc × Large White_old × Landrace_old); DLWLR2 (Duroc × Large White_new × Landrace_new). Blue line represents female pig, yellow line represents male pig.

Effects of genotype and sex on carcass traits are shown in Table 2. When a significant interaction effect was present, main effects were not further compared, and the results are presented in Figure 1. Genotype and sex had no significant effect on live weight, hot or cold carcass weight, or bone percentage (Table 2). However, significant genotype effects were observed for lean percentage, fat percentage, and lean-to-fat ratio. LWLR1 pigs had the lowest lean percentage and lean-to-fat ratio (*p* < 0.05) compared to other genotypes, except LWLR2, and the highest fat percentage compared to D and DLWLR2 pigs (*p* < 0.05).

As shown in Figure 1, genotype × sex interaction significantly influenced hot and cold dressing percentages, purge loss, bone percentage, and carcass length (*p* < 0.05). Dressing percentages were higher in gilts than barrows in all genotypes except D and LWLR1. Carcass length and bone percentage were significantly higher in gilts than barrows only in the Duroc group. DLWLR1 gilts exhibited lower purge loss compared to DLWLR1 barrows (*p* < 0.05), while no sex-related differences were observed in other genotypes.

### 3.2. Myosin Heavy Chain Isoform Expression

Sex had no significant effect on muscle fiber type distribution corresponding to MyHC isoform expression (Table 3). MyHC I expressed higher in LWLR2 and DLWLR2 pigs (*p* < 0.05) than in other genotypes, while MyHC IIA expressed significantly lower in D and DLWLR1 pigs. Duroc pigs had the highest percentage of MyHC IIX expression, whereas MyHC IIB expression was greater in LWLR1 and DLWLR1 pigs, and lowest in D pigs (*p* < 0.05). No genotype × sex interaction was detected for MyHC isoforms.

### 3.3. Meat Characteristics

**Figure 2:**
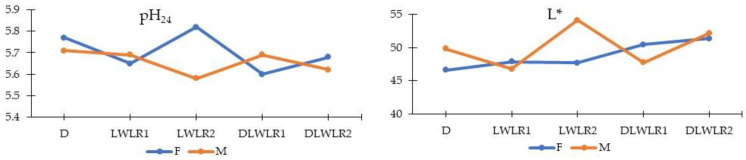
Interaction between genotypes and sexes on meat pH_24_ and L* (lightness) value in pigs. D (Duroc × Duroc); LWLR1 (Large White_old ×Landrace_old); LWLR2 (Large White_new × Landrace_new); DLWLR1 (Duroc × Large White_old × Landrace_old); DLWLR2 (Duroc × Large White_new × Landrace_new). Blue line represents female pig, yellow line represents male pig.

As shown in Table 4, Duroc pigs had significantly lower pH_45_ than other genotypes, except DLWLR1 (*p* < 0.05). Genotype × sex interaction significantly affected pH_24_ and L*. LWLR2 gilts showed higher pH_24_ values than their barrow counterparts, while barrows exhibited greater lightness than gilts in the D and LWLR2 groups (Figure 2). Among genotypes, LWLR1 pigs had the lowest yellowness (b*) values. No significant effects were found for a* (redness), sarcomere length, fiber diameter, or drip loss.

DLWLR2 pigs had the lowest thawing loss on day 1, comparable only to D pigs (*p* < 0.05), while thawing loss on day 5 was highest in DLWLR1 and LWLR1 pigs. Cooking loss on day 1 was greater in D and DLWLR1 pigs than others, while on day 5, DLWLR1 had the highest cooking loss. On day 1, D pigs had lower shear force than other genotypes (*p* < 0.05), except DLWLR1; this pattern reversed by day 5, when DLWLR1 and LWLR1 showed the highest shear force values.

### 3.4. Chemical Composition and Ribonucleotides

Ether extract (IMF) content was highest in D pigs and lowest in LWLR1 (*p <* 0.05), while crude protein was greatest in LWLR1 pigs, followed by LWLR2 and DLWLR1 (Table 5). Ash content was highest in DLWLR1 pigs (*p* < 0.05). Sex had no significant effect on chemical composition.

Genotype significantly influenced inosine, IMP, and GMP content, but not hypoxanthine (Table 5). LWLR1 pigs had the highest inosine and GMP concentrations, while D pigs showed the lowest IMP levels. DLWLR2 pigs had lower inosine than other genotypes, except LWLR2. No significant effects of sex or genotype × sex interaction were detected.

### 3.5. Calpain System Gene Expression

The expression levels of calpain system genes in LL muscle varied significantly by genotype (Table 6). DLWLR2 pigs had the highest *CAPN1* expression, while DLWLR1 and LWLR1 pigs had the lowest. *CAPN2* expression was higher in DLWLR2 and LWLR2 pigs, and lowest in Duroc pigs. *CAST* expression was highest in LWLR2 pigs, comparable only to DLWLR2. No sex or interaction effects were observed.

### 3.6. Fatty Acids

**Figure 3:**
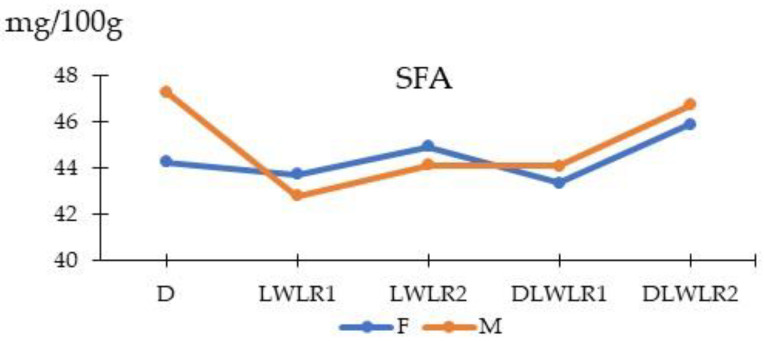
Interaction between genotypes and sexes on saturated fatty acid (SFA) in pigs. D (Duroc × Duroc); LWLR1 (Large White_old ×Landrace_old); LWLR2 (Large White_new × Landrace_new); DLWLR1 (Duroc × Large White_old × Landrace_old); DLWLR2 (Duroc × Large White_new × Landrace_new). Blue line represents female pig, yellow line represents male pig.

As presented in Table 7, genotype significantly influenced the concentration of most fatty acids, total polyunsaturated fatty acids (PUFAs), and the P:S (PUFA:SFA) ratio. LWLR1 pigs had the highest PUFA (C18:2n-6, C18:3n-3), P:S ratio, and the lowest C14:0. C15:0 was most abundant in the two-way crossbreds, while D and DLWLR2 pigs had the highest C18:0 content. LWLR2 pigs exhibited the highest C16:1 concentration. A significant genotype × sex interaction (*p* < 0.05) was found for SFA, with Duroc barrows showing higher SFA levels than gilts (Figure 3). Sex had no effect on individual fatty acid proportions except for C14:0 and C18:0.

### 3.7. Principal Component Analysis

**Figure 4:**
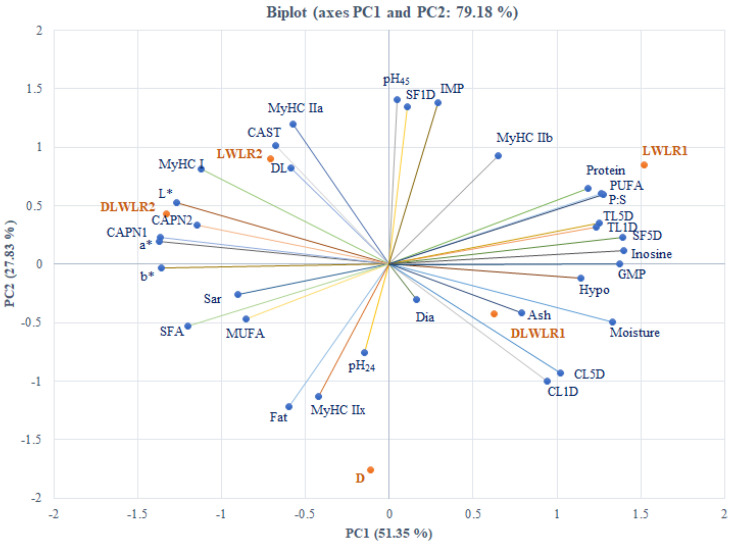
Principal component analysis (PCA) biplot illustrating the relationships among meat quality traits, chemical composition, and the expression of myosin heavy chain isoforms and calpain system genes across different pig genotypes. The colored lines represent loading vectors of individual variables. L* = lightness, a* = redness, b* = yellowness, SFD1 = shear force at day 1 postmortem, MyHC IIb = myosin heavy chain IIb, CL = cooking loss, DL = drip loss, TL = thawing loss, Dia = muscle fiber diameter, Sar = sarcomere length, Hypo = hypoxanthine, IMP = inosine monophosphate, GMP = guanosine monophosphate, SFA = saturated fatty acids, MUFA = monounsaturated fatty acids, PUFA = polyunsaturated fatty acids, P:S = PUFA: SFA. D, Duroc × Duroc; LWLR1, Large White_old × Landrace_old; LWLR2, Large White_new × Landrace_new; DLWLR1, Duroc × LWLR1; DLWLR2, Duroc × LWLR2.

The PCA revealed that the first two principal components (PC1 and PC2) accounted for 79.18% of the total variance in the data, with PC1 explaining 51.35% and PC2 explaining 27.83% (Figure 4). PC1 primarily represented physicochemical meat quality traits, while PC2 captured variations related to muscle fiber composition. In the PCA biplot, moisture, protein, thawing loss (days 1 and 5), cooking loss (day 5), shear force (day 5), PUFA, P:S ratio, and nucleotide compounds (hypoxanthine, inosine, GMP) were clustered on the right side of PC1, suggesting their close association. In contrast, L*, a*, b*, MyHC I expression, *CAPN1*, *CAPN2*, and SFA were aligned on the left side, indicating an opposing trend. For PC2, traits such as pH_45_, shear force on day 1, MyHC IIA and IIB, and *CAST* expression were positioned on one side, while cooking loss on day 1, %fat, and MyHC IIX were located on the opposite end, illustrating an inverse relationship between oxidative and glycolytic muscle properties. Genotype clustering showed that purebred Duroc pigs were positively associated with higher IMF and pH_24_, and negatively with pH_45_ and shear force on day 1. DLWLR1 and LWLR1 genotypes clustered with high protein content, GMP, and PUFA, suggesting better nutritional and biochemical profiles. Meanwhile, DLWLR2 and LWLR2 were associated with favorable color traits (L*, a*, b*) and oxidative fiber characteristics, reflecting improved visual and structural meat quality.

## 4. Discussion

The influence of genotype and sex on carcass traits, muscle fiber composition, and meat quality has been widely documented in pigs [11,12,13,15]. However, data specific to exotic breeds under Thai production conditions remain limited. This study contributes valuable insights by comparing purebred Duroc, two-way crossbred (LWLR1, LWLR2), and three-way crossbred (DLWLR1, DLWLR2) pigs, with a focus on maternal line variation.

Despite the genetic variation, no significant differences were observed in live weight or carcass weight across genotypes and sexes, likely due to standardized slaughter weights. However, clear differences in carcass composition were identified. LWLR1 pigs exhibited higher fat and lower lean percentages compared with the other genotypes, resulting in the lowest lean-to-fat ratio. These outcomes may stem from differences in genetic selection for fat deposition, energy metabolism, and growth efficiency, especially given that LWLR1 represents an older maternal line with less emphasis on lean growth.

Although the main effect of sex was limited, females tended to exhibit a higher lean/fat ratio than males. Normally, testosterone in intact males promotes muscle accretion and reduces fat deposition [25]; however, in this study, males were castrated at one week of age. The absence of testosterone likely contributed to the lower proportion of lean meat and higher fat deposition in castrated males compared with females. Consistently, Lertpatarakomol et al. [26] and Peinado et al. [27] reported a lower loin percentage and a higher fat percentage in castrated males than in female pigs.

Genotype × sex interactions were evident in dressing percentages, carcass length, bone percentage, and purge loss. Dressing percentages were higher in gilts than barrows in all genotypes except Duroc and LWLR1, aligning with prior studies showing higher lean deposition in gilts that contributes to heavier carcasses post-dressing [14,28]. The increased carcass length and bone percentage in Duroc gilts compared to barrows suggest sex-dependent skeletal growth patterns specific to this genotype. Additionally, the lower purge loss observed in DLWLR1 gilts compared to barrows may reflect differences in muscle composition, water-holding capacity, or fat distribution that are sex-specific and more pronounced in this particular cross.

Muscle fiber composition varied significantly among genotypes, with Duroc pigs showing higher MyHC IIX and lower MyHC IIB expression, indicative of a glycolytic profile favoring intramuscular fat accumulation. In contrast, LWLR1 and DLWLR1 pigs exhibited higher MyHC IIB, which correlates with rapid glycolytic metabolism and lower oxidative capacity, potentially explaining their higher shear force and reduced tenderness. LWLR2 and DLWLR2 pigs had elevated MyHC I and IIA, associated with better oxidative capacity and possibly improved endurance and meat quality traits. Sex had no effect on muscle fiber types, indicating similar growth rates between gilts and castrated males. This is consistent with the results of studies by Kim et al. [29] and Jeong et al. [30] that found no gender-related differences in muscle fiber types in both purebred and crossbred pigs.

Meat quality traits such as pH, water-holding capacity, and tenderness were strongly influenced by genotype. Duroc pigs had the lowest initial pH (pH_45_), which could accelerate postmortem glycolysis and proteolysis, contributing to lower shear force and greater tenderness on day 1. The lower pH_45_ in the purebred Duroc pigs, compared to other genotypes except DLWLR1, may be attributed to the lower concentrations of MyHC I and MyHC IIA fibers and the predominance of glycolytic MyHC IIX fibers. These fibers produce more lactic acid, resulting in a lower pH [31]. Thawing and cooking losses were lowest in DLWLR2 and LWLR2 pigs, suggesting better structural integrity and water-holding capacity in these genotypes. Over time, meat tenderness in DLWLR1 and LWLR1 pigs was lower than in the other groups, which may be associated with their higher proportion of MyHC IIB fibers and lower expression of the *CAPN1* gene. MyHC IIB fibers are glycolytic and typically linked to larger fiber diameters and tougher meat texture [32,33], while reduced *CAPN1* expression suggests lower calpain activity, potentially limiting postmortem proteolysis and reducing tenderization during aging.

Calpains are calcium-activated cysteine proteases involved in postmortem meat tenderization through the degradation of myofibrillar and cytoskeletal proteins [34]. *CAPN1* and *CAPN2* encode μ-calpain and m-calpain, respectively, which differ in calcium sensitivity [35]. *CAST* encodes calpastatin, a specific inhibitor that regulates calpain activity [35,36]. In this study, DLWLR2 and LWLR2 pigs showed higher *CAPN1*, *CAPN2*, and *CAST* mRNA levels, suggesting greater proteolytic potential. This may enhance tenderness due to increased postmortem protein degradation. The upregulation of calpain genes in these genotypes may be associated with a higher proportion of oxidative and oxido-glycolytic fibers (MyHC I and IIA), which support calpain stability via higher muscle pH. Elevated *CAST* expression may act as a regulatory response to prevent excessive proteolysis. These results suggest that genotype and muscle fiber characteristics modulate the calpain system, with implications for meat quality.

Fatty acid composition plays a crucial role in meat flavor, texture, nutrition, and health value [37,38]. In this study, sex had no effect on the fatty acid profile of the LL muscle, whereas genotype significantly influenced most individual fatty acids, except C18:1n-9. Purebred Duroc pigs exhibited the highest IMF content and a greater proportion of MyHC IIX fibers. In contrast, LWLR1 pigs showed elevated levels of C18:2n-6, C18:3n-3, PUFA, and a higher P:S ratio. This lipid profile may be associated with a higher phospholipid-to-triacylglycerol ratio, likely resulting from the lower fat content in LWLR1 pigs. As phospholipids are relatively stable components of muscle cell membranes, while triacylglycerol levels decrease with reduced IMF, leaner muscle tends to exhibit a proportionally higher phospholipid contribution [39]. Their lower C18:1n-9 (oleic acid) levels (*p* = 0.08) may result from PUFA-mediated suppression of stearoyl-CoA desaturase (SCD) activity. SCD converts stearic acid (C18:0) to oleic acid, but its expression is downregulated by C18:2n-6 and C18:3n-3 through feedback inhibition [40,41]. Higher concentrations of C14:0, C16:0, and C18:0 in Duroc and DLWLR1 pigs likely reflect greater triacylglycerol content, while elevated C16:1 in LWLR2 pigs suggests increased SCD activity. Total MUFA, PUFA, and SFA levels corresponded to the distribution of individual fatty acids. A significant genotype × sex interaction was observed for SFA, with Duroc barrows showing higher levels than gilts, possibly due to sex-related fat metabolism. This effect was less pronounced in crossbreds, likely due to genetic variability and heterosis.

Ribonucleotides, particularly IMP and GMP, contribute significantly to meat flavor. As adenosine triphosphate (ATP) and guanosine triphosphate (GTP) degrade postmortem, they produce flavor-enhancing compounds like IMP and GMP [42]. Over time, IMP degrades into inosine and then hypoxanthine. While IMP and GMP provide umami flavor, inosine is tasteless, and hypoxanthine has a bitter taste [42,43]. In this study, IMP was the predominant nucleotide, a finding consistent with previous studies in pigs [31,44]. The higher inosine and GMP levels in LWLR1 pigs may be related to their higher proportion of MyHC IIB fibers and moderate levels of MyHC IIX fibers. The postmortem pH plays a crucial role in the stability and degradation of ribonucleotides, as pH affects the enzymes involved in ATP breakdown [42]. Fast-twitch muscle fibers, which undergo rapid postmortem glycolysis, tend to have a lower pH, accelerating nucleotide degradation [42].

The PCA provided a comprehensive view of the relationships among studied traits. Most parameters group within PC1, associated with physicochemical meat properties, while PC2 captures variation in muscle fiber types. The LL muscles characterized by high moisture and protein levels, thawing loss on both day 1 and day 5, cooking loss on day 5, shear force on day 5, PUFA, P:S, hypoxanthine, inosine, and GMP exhibit features that contrast those showing higher lightness, redness, yellowness, MyHC I, *CAPN1*, *CAPN2*, and SFA. For PC2, which captures the nuances in muscle fiber characteristics, pH_24_, shear force on day 1, MyHC IIA and MyHC IIX, and *CAST* expression levels were positioned opposite to cooking loss on day 1, IMF, and MyHC IIX. This contrast reflects how muscle fiber composition influence meat quality in different genotypes. The biplot reveals that the purebred Duroc pigs were positively associated with higher IMF and pH_24_, but negatively correlated with pH_45_ and shear force on day 1. In contrast, the DLWLR1 and LWLR1 genotypes were more closely linked with high protein content, PUFA, and GMP, indicating superior nutritional and biochemical properties. On the other hand, DLWLR2 and LWLR2 genotypes were more associated with color parameters (L*, a*, b*) and muscle fiber traits, suggesting distinct visual and structural qualities in their meat. This comprehensive analysis highlights how different pig genotypes exhibit unique combinations of meat quality traits.

### Limitations

The relatively small sample size used in this study was determined by ethical and budgetary considerations. While sufficient to detect meaningful differences among genotypes and sexes, a larger sample size would provide greater statistical power and further strengthen the generalizability of the findings.

## 5. Conclusions

This study evaluated the effects of genotype and sex on carcass traits, muscle fiber composition, and meat quality of pigs under Thai production conditions, with emphasis on maternal line contributions in Duroc crossbreeding. Genotype, more than sex, significantly affected carcass composition, MyHC isoform expression, and multiple meat quality parameters. Purebred Duroc pigs showed higher intramuscular fat and tenderness but also a greater proportion of saturated fatty acids, while the old maternal line LWLR1 exhibited superior protein, PUFAs, and flavor-related nucleotide levels. The new maternal line LWLR2 and its Duroc cross (DLWLR2) displayed oxidative muscle traits, improved water-holding capacity, and elevated proteolysis-related gene expression. These findings underscore the role of maternal line selection in optimizing pork quality and offer practical guidance for breeding programs targeting meat quality improvement in tropical production systems.

## Figures and Tables

**Table 1:** List of primers used to quantify genes.

Gene *	Primer Sequence (5′ to 3′)		Annealing Temperature (°C)	Product Size (bp)	Accession No.	Reference
*MYH7*	Forward	AAGGGCTTGAACGAGGAGTAGA	60	115	AB053226	[16]
	Reverse	TTATTCTGCTTCCTCCAAAGGG				
*MYH2*	Forward	GCTGAGCGAGCTGAAATCC	60	137	AB025260	
	Reverse	ACTGAGACACCAGAGCTTCT				
*MYH1*	Forward	AGAAGATCAACTGAGTGAACT	57	149	AB025262	
	Reverse	AGAGCTGAGAAACTAACGTG				
*MYH4*	Forward	ATGAAGAGGAACCACATTA	55	166	AB025261	
	Reverse	TTATTGCCTCAGTAGCTTG				
*CAPN1*	Forward	GACACCCTCCTGCACCGA	55	101	AF263610	[17]
	Reverse	TCCACCCACTCCCCAAACT				
*CAPN2*	Forward	ACATGCACACCATCGGCTTT	55	101	U01181	
	Reverse	CGCTCTGTGCGTCAGGAAG				
*CAST*	Forward	AGGCTGTAAAAACAGAACCTG	55	201	M20160	[18]
	Reverse	ATTTCTCTGATGTTGGCTGCTC				
*GAPDH*	Forward	TCACTGCCACCCAGAAGA	65	229	ABO38240	
	Reverse	TACCAGGAAATGAGCTTGAC				

* Genes *MYH7*, *MYH2*, *MYH1*, and *MYH4* encode the myosin heavy chain isoforms MyHC I, MyHC IIA, MyHC IIX, and MyHC IIB, respectively. *CAPN1*, calpain 1; *CAPN2*, calpain 2; *CAST*, calpastatin; *GAPDH*, glyceraldehyde-3-phosphate dehydrogenase.

**Table 2:** Carcass traits in pigs from different genotypes and sexes.

Trait	Genotype (G)					Sex (S)		RMSE	*p*-value		
	D	LWLR1	LWLR2	DLWLR1	DLWLR2	F	M		G	S	G × S
Live Weight (kg)	112.20	110.80	108.50	110.70	112.40	110.84	111.00	3.83	0.178	0.883	0.656
Hot Carcass (kg)	87.14	86.62	84.84	85.45	85.86	86.62	85.34	3.17	0.511	0.160	0.241
% Hot Carcass	77.66	78.18	78.18	77.18	76.41	78.15	76.89	1.02	0.001	<0.0001	0.000
Cold Carcass (kg)	85.32	84.60	82.35	83.41	83.34	84.44	83.17	3.04	0.231	0.146	0.223
% Cold Carcass	76.04	76.36	75.89	75.35	74.17	76.18	74.94	0.96	<0.0001	<0.0001	<0.0001
%Purge Loss	2.08	2.32	2.75	2.37	2.93	2.47	2.52	0.22	<0.0001	0.410	0.024
% Lean	44.71 ^a^	42.25 ^b^	45.67 ^a^	44.94 ^a^	45.62 ^a^	44.89	44.25	1.57	<0.0001	0.172	0.501
% Fat	10.86 ^b^	12.71 ^a^	11.82 ^ab^	11.13 ^ab^	10.88 ^b^	11.27	11.69	1.11	0.002	0.184	0.075
% Bone	8.05	7.87	8.05	7.78	8.18	7.99	7.98	0.29	0.034	0.971	0.027
Lean/Fat	4.19 ^a^	3.34 ^b^	3.89 ^ab^	4.07 ^a^	4.25 ^a^	4.08	3.92	0.52	0.002	0.096	0.126
Carcass Length (cm)	85.70	85.00	81.50	84.10	83.00	84.20	83.52	1.82	<0.0001	0.194	0.017

^a, b^ Least squares means within a row with different superscripts differ (*p* < 0.05). Superscripts are shown only when the genotype × sex interaction was not significant; when the interaction was significant, main effects were not compared. RMSE, Root mean square error. D, Duroc × Duroc; LWLR1, Large White_Old × Landrace_Old; LWLR2, Large White_new ×Landrace_new; DLWLR1, Duroc × LWLR1; DLWLR2, Duroc × LWLR2.

**Table 3:** Myosin heavy chain isoform expression^1^ of *Longissimus lumborum* of pigs from different genotypes and sexes.

Trait	Genotype (G)					Sex (S)		RMSE	*p*-Value		
	D	LWLR1	LWLR2	DLWLR1	DLWLR2	F	M		G	S	G × S
MyHC I	0.16 ^b^	0.17 ^b^	1.28 ^a^	0.12 ^b^	1.09 ^a^	0.58	0.55	0.39	<0.0001	0.979	0.183
MyHC IIA	2.71 ^b^	4.11 ^a^	4.11 ^a^	2.55 ^b^	4.37 ^a^	3.67	3.65	0.95	<0.0001	0.786	0.118
MyHC IIX	70.4 ^a^	32.67 ^c^	47.30 ^b^	38.38 ^bc^	39.68 ^bc^	47.59	43.79	12.28	<0.0001	0.212	0.343
MyHC IIB	26.9 ^c^	67.27 ^a^	46.25 ^b^	64.96 ^a^	55.03 ^ab^	50.46	53.71	13.85	<0.0001	0.374	0.269

^a, b, c^ Least squares means within a row with different superscripts differ (*p* < 0.05). Superscripts are shown only when the genotype × sex interaction was not significant; when the interaction was significant, main effects were not compared. RMSE, Root mean square error. D, Duroc × Duroc; LWLR1, Large White_Old × Landrace_Old; LWLR2, Large White_new × Landrace_new; DLWLR1, Duroc × LWLR1; DLWLR2, Duroc × LWLR2. ^1^ relative expression ratio.

**Table 4:** Meat characteristics of *Longissimus lumborum* of pigs from different genotypes and sexes.

Trait	Genotype (G)					Sex (S)		RMSE	*p*-Value		
	D	LWLR1	LWLR2	DLWLR1	DLWLR2	F	M		G	S	G × S
pH_45_	6.36 ^c^	6.80 ^a^	6.77 ^ab^	6.54 ^bc^	6.71 ^ab^	6.66	6.61	0.27	0.003	0.560	0.970
pH_24_	5.74	5.67	5.70	5.65	5.65	5.70	5.66	0.10	0.209	0.093	0.008
Lightness (L*)	48.22	47.34	50.91	49.09	51.79	48.80	50.14	2.28	0.000	0.047	0.001
Redness (a*)	7.90	7.33	8.39	7.91	8.69	8.22	7.87	1.51	0.108	0.291	0.602
Yellowness (b*)	1.64 ^a^	0.63 ^b^	2.12 ^a^	1.50 ^a^	2.19 ^a^	1.49	1.74	0.80	0.001	0.275	0.235
Sarcomere Length (u)	1.76	1.72	1.74	1.78	1.82	1.74	1.79	0.11	0.351	0.136	0.650
Muscle Fiber Diameter (u)	73.73	72.84	74.82	74.33	71.16	74.21	72.55	7.75	0.848	0.473	0.922
Drip Loss (%)	2.01	2.22	3.35	2.77	2.62	2.55	2.64	0.94	0.057	0.744	0.052
Thawing Loss Day 1 (%)	5.33 ^bc^	8.44 ^a^	6.65 ^ab^	8.24 ^a^	3.72 ^c^	6.50	6.46	2.34	0.000	0.951	0.934
Thawing Loss Day 5 (%)	7.02 ^b^	10.09 ^a^	7.53 ^b^	9.93 ^a^	7.17 ^b^	8.08	8.61	2.42	0.010	0.455	0.771
Cooking Loss Day 1 (%)	24.45 ^a^	22.65 ^ab^	20.26 ^bc^	24.89 ^a^	18.91 ^c^	22.68	21.79	2.75	0.000	0.286	0.121
Cooking Loss Day 5 (%)	22.74 ^ab^	21.24 ^b^	18.63 ^c^	23.08 ^a^	17.16 ^c^	20.88	20.26	1.91	<0.0001	0.278	0.103
Shear Force Day 1 (kg)	4.56 ^c^	6.01 ^a^	5.92 ^a^	4.90 ^bc^	5.33 ^ab^	5.31	5.37	0.76	0.001	0.791	0.127
Shear Force Day 5 (kg)	4.48 ^b^	5.67 ^a^	4.40 ^b^	5.09 ^a^	4.15 ^b^	4.88	4.64	0.63	<0.0001	0.201	0.135

^a, b, c^ Least squares means within a row with different superscripts differ (*p <* 0.05). Superscripts are shown only when the genotype × sex interaction was not significant; when the interaction was significant, main effects were not compared. RMSE, Root mean square error. D, Duroc × Duroc; LWLR1, Large White_Old × Landrace_Old; LWLR2, Large White_new × Landrace_new; DLWLR1, Duroc × LWLR1; DLWLR2, Duroc × LWLR2. L* represents lightness (0 = black, 100 = white), a* represents the red–green axis (positive values = red, negative values = green), and b* represents the yellow–blue axis (positive values = yellow, negative values = blue).

**Table 5:** Chemical composition and ribonucleotide content in *Longissimus lumborum* muscle of pigs from different genotypes and sexes.

Trait	Genotype (G)					Sex (S)		RMSE	*p*-Value		
	D	LWLR1	LWLR2	DLWLR1	DLWLR2	F	M		G	S	G × S
Chemical Composition (%)											
Moisture	73.86	74.13	73.02	73.97	72.77	73.65	73.45	1.22	0.111	0.620	0.760
Ether Extract	4.02 ^a^	1.69 ^d^	1.95 ^cd^	2.37 ^bc^	3.00 ^b^	2.46	2.75	0.734	<0.0001	0.169	0.285
Crude Protein	22.94 ^cd^	24.46 ^a^	23.66 ^b^	23.62 ^bc^	22.56 ^d^	23.61	23.29	0.80	<0.0001	0.163	0.570
Ash	1.17 ^b^	1.16 ^bc^	1.17 ^b^	1.25 ^a^	1.10 ^c^	1.18	1.16	0.07	0.003	0.359	0.187
Ribonucleotide (mg/100 g)											
Hypoxanthine	8.80	9.64	7.96	8.55	8.39	8.69	8.64	1.36	0.098	0.900	0.516
Inosine	49.00 ^c^	66.00 ^a^	44.76 ^cd^	58.47 ^b^	42.39 ^d^	51.43	52.82	6.72	<0.0001	0.475	0.412
IMP	360.3 ^c^	444.96 ^a^	438.40 ^ab^	399.14 ^bc^	410.56 ^ab^	405.62	415.72	49.55	0.004	0.481	0.844
GMP	4.17 ^b^	5.10 ^a^	3.66 ^c^	4.48 ^b^	3.74 ^c^	4.27	4.19	0.40	<0.0001	0.503	0.747

^a, b, c, d^ Least squares means within a row with different superscripts differ (*p* < 0.05). Superscripts are shown only when the genotype × sex interaction was not significant; when the interaction was significant, main effects were not compared. RMSE, Root mean square error. D, Duroc × Duroc; LWLR1, Large White_Old × Landrace_Old; LWLR2, Large White_new × Landrace_new; DLWLR1, Duroc × LWLR1; DLWLR2, Duroc × LWLR2; IMP, inosine monophosphate; GMP, guanosine monophosphate.

**Table 6:** Expression levels^1^ of *CAPN1*, *CAPN2*, and *CAST* genes in *Longissimus lumborum* muscle of pigs from different genotypes and sexes.

Trait	Genotype (G)					Sex (S)		RMSE	*p*-Value		
	D	LWLR1	LWLR2	DLWLR1	DLWLR2	F	M		G	S	G × S
*CAPN1*	0.62 ^c^	0.34 ^d^	0.79 ^b^	0.36 ^d^	1.08 ^a^	0.61	0.66	0.16	<0.0001	0.299	0.800
*CAPN2*	0.87 ^c^	0.84 ^bc^	1.24 ^ab^	1.10 ^bc^	1.49 ^a^	1.03	1.18	0.40	0.006	0.184	0.853
*CAST*	0.82 ^b^	0.93 ^b^	1.30 ^a^	0.75 ^b^	1.01 ^ab^	0.98	0.94	0.32	0.008	0.718	0.944

^a, b, c, d^ Least squares means within a row with different superscripts differ (*p* < 0.05). Superscripts are shown only when the genotype × sex interaction was not significant; when the interaction was significant, main effects were not compared. RMSE, Root mean square error. D, Duroc × Duroc; LWLR1, Large White_Old × Landrace_Old; LWLR2, Large White_new × Landrace_new; DLWLR1, Duroc × LWLR1; DLWLR2, Duroc ×LWLR2. ^1^ Expression of target genes, *CAPN1* (calpain 1), *CAPN2* (calpain 2), *CAST* (calpastatin) were normalized to *GAPDH* (glyceraldehyde-3-phosphate dehydrogenase).

**Table 7:** Fatty acid composition (% total fatty acids) of *Longissimus lumborum* muscle of pigs from different genotypes and sexes.

Trait	Genotype (G)					Sex (S)		RMSE	*p*-Value		
	D	LWLR1	LWLR2	DLWLR1	DLWLR2	F	M		G	S	G × S
C14:0	1.67 ^a^	1.37 ^c^	1.60 ^ab^	1.47 ^bc^	1.65 ^a^	1.60	1.50	0.16	0.0005	0.039	0.561
C15:0	0.63 ^c^	1.57 ^a^	1.37 ^a^	0.97 ^b^	0.92 ^bc^	1.15	1.04	0.36	<0.0001	0.302	0.573
C16:0	27.66 ^ab^	25.46 ^d^	26.78 ^bc^	26.30 ^cd^	27.81 ^a^	26.76	26.85	0.95	<0.0001	0.751	0.143
C16:1	3.70 ^b^	3.48 ^b^	4.45 ^a^	3.80 ^b^	3.44 ^b^	3.84	3.70	0.71	0.021	0.498	0.569
C18:0	14.53 ^a^	11.72 ^c^	11.88 ^c^	13.02 ^b^	14.19 ^a^	12.58	13.55	1.08	<0.0001	0.003	0.067
C18:1n9c	41.53	39.80	41.29	42.25	41.92	41.17	41.55	2.00	0.083	0.506	0.104
C18:2n6c	7.85 ^bc^	12.01 ^a^	8.66 ^bc^	9.15 ^b^	7.25 ^c^	9.36	8.61	1.88	<0.0001	0.166	0.371
C20:1	0.80 ^ab^	0.96 ^a^	0.68 ^b^	0.68 ^b^	0.72 ^b^	0.78	0.75	0.18	0.005	0.534	0.527
C18:3n3	0.33 ^b^	0.49 ^a^	0.40 ^ab^	0.38 ^b^	0.35 ^b^	0.40	0.37	0.10	0.014	0.333	0.698
C23:0	1.29 ^b^	3.13 ^a^	2.88 ^a^	1.97 ^b^	1.72 ^b^	2.34	2.05	0.84	<0.0001	0.231	0.311
ΣMUFA	46.03	44.25	46.42	46.74	46.08	45.80	46.00	2.11	0.097	0.728	0.088
ΣPUFA	8.18 ^bc^	12.49 ^a^	9.06 ^bc^	9.53 ^b^	7.60 ^c^	9.76	8.98	1.96	<0.0001	0.169	0.387
ΣSFA	45.78	43.26	44.53	43.73	46.31	44.44	45.01	1.27	<0.0001	0.121	0.007
P:S	0.18 ^bc^	0.29 ^a^	0.20 ^bc^	0.22 ^b^	0.16 ^bc^	0.22	0.20	0.04	<0.0001	0.142	0.399

^a, b, c, d^ Least squares means within a row with different superscripts differ (*p* < 0.05). Superscripts are shown only when the genotype × sex interaction was not significant; when the interaction was significant, main effects were not compared. RMSE, Root mean square error. D, Duroc × Duroc; LWLR1, Large White_Old × Landrace_Old; LWLR2, Large White_new × Landrace_new; DLWLR1, Duroc × LWLR1; DLWLR2, Duroc × LWLR2. ΣSFA, total saturated fatty acids (c14:0 + C15:0 + c16:0 + c18:0 + c23:0). ΣPUFA, total polyunsaturated fatty acids, (c18:2n6c + c18:3n3). ΣMUFA, total monounsaturated fatty acids (c16:1 + c18:1n9c + c20:1). P:S, (c18:2n6c + c18:3n3)/ (c14:0 + C15:0 + c16:0 + c18:0 + c23:0).

## Data Availability

The data that support the findings of this study are available upon reasonable request.
